# Condyloma acuminata in the tongue and palate of a sexually abused child: a case report

**DOI:** 10.1186/1756-0500-7-467

**Published:** 2014-07-23

**Authors:** Ana Clélia Cânovas Percinoto, Marcelle Danelon, Marcelo Macedo Crivelini, Robson Frederico Cunha, Célio Percinoto

**Affiliations:** 1 Araçatuba Dental School, Univ. Estadual Paulista (UNESP), Rua José Bonifácio 1193, 16015-050 Araçatuba, SP, Brazil; 2 East Paulista University, Presidente Prudente, SP, Brazil

**Keywords:** Condyloma acuminata, Child, Sexual abuse, Dentistry

## Abstract

**Background:**

Condyloma acuminata caused by human papilloma viruses, (HPV) is a sexually transmitted disease (STD) appearing most frequently as soft, pink cauliflower like growths in moist areas, such as the genitalia, mouth and other places. The disease is highly contagious, can appear singly or in groups, small or large. In children, the isolation of a sexually transmitted organism may be the first indication that an abuse has occurred. Although the presence of a sexually transmissible agent from a child beyond the neonatal period is suggestive of sexual abuse, exceptions do exist.

**Case presentation:**

The authors report the clinical case of a five-year-old Caucasian male with lesions located in the dorsal surfaces of the posterior tongue and palate. Both lesions had a firm consistency, reddish appearance and presence of whitish areas and regions of ulceration. During the interview, the mother reported that the boy had been sexually abused.

**Conclusion:**

Sexually transmitted disease may occur during sexual abuse. Dentists as well as pediatricians have a role to play in identifying and treating these children. The diagnosis is essentially clinical (anamnesis and physical examination), but also the use of cytology eventually resorts to biopsy of the suspicious lesions for histological examination. The therapeutic option was the excision of the lesions.

## Background

HPV is the acronym used to identify the human papillomavirus, responsible for condyloma acuminata [1], which affects the genitalia, perianal region, rectal and urethral mucosae, and sometimes the oral cavity [2]. Both sexes are affected equally, and the infection is usually transmitted by sexual intercourse [3]. The peak incidence occurs in people in their 20s, and reports of condyloma acuminata in children are rare [4].

The evaluation of the child with condyloma acuminata requires a thorough medical and social evaluation to determine whether there is any evidence of sexual abuse or other sexually transmitted disease (STD), and to establish the source of the virus [5,6].

Sexual abuse has been considered the main mode of transmission and some authors consider that the mere presence of warts in children may be an indication that there is evidence of sexual abuse [7]. Denunciations of abuse against children have been frequent and constitute a serious public health problem. The topic is uncomfortable for many doctors and dentists due to insufficient training, and/or through ignorance of the dimensions of the problem. Suspicion of abuse can have significant indirect elements: explanations of the injury found are vague or absent, versions of the facts differ from one moment to another and the history is inconsistent with the physical findings [8,9].

Physicians and dentists are usually the first professionals to observe and recognize signs of non-accidental or intentional injury. Sexual abuse of children must be reported to the Child Protection Council, preferably directly to the Child Abuse Team. The decision to prosecute depends on the nature of the offence, the offender’s attitude, the availability of treatment, and the evidence available [10,11].

Given that STD can be transmitted during a sexual assault, this paper aims to report a case of condyloma acuminata on a child’s tongue and palate, as well as the involved clinical practice.

## Case presentation

**Figure 1:**
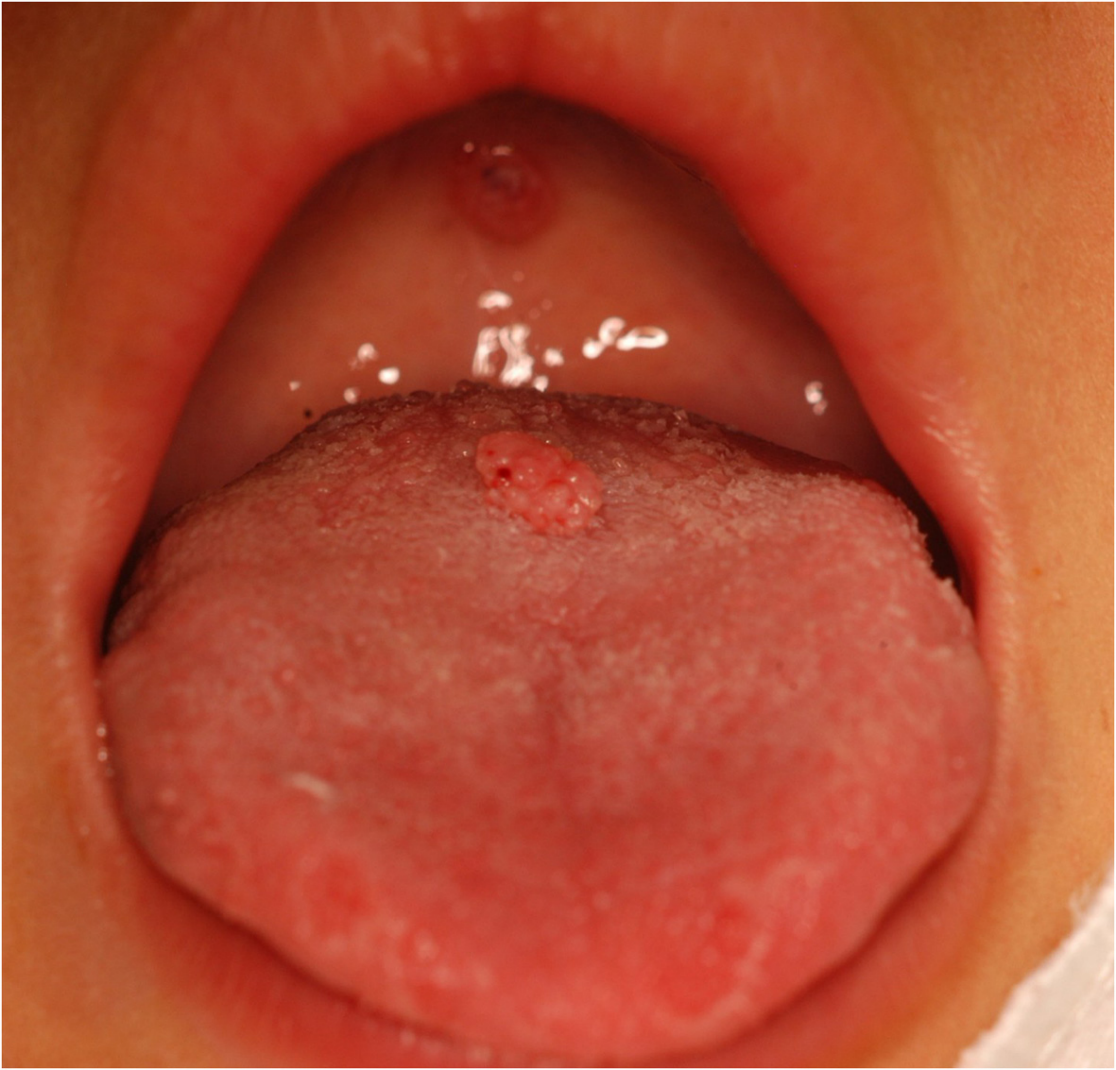
Lesion located on the tongue with a pedicled base.

**Figure 2:**
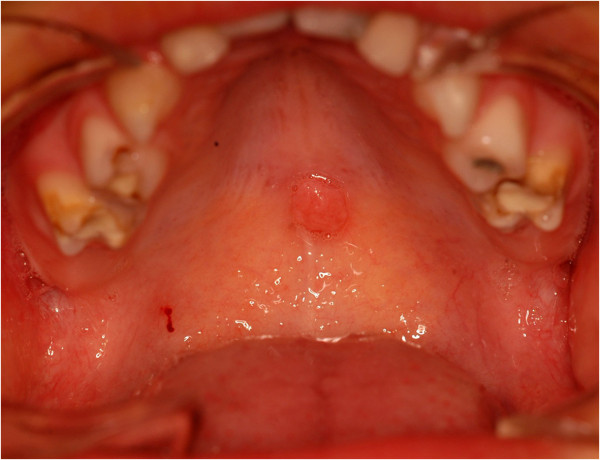
Lesion located in the posterior area of the hard palate with sessile base.

**Figure 3:**
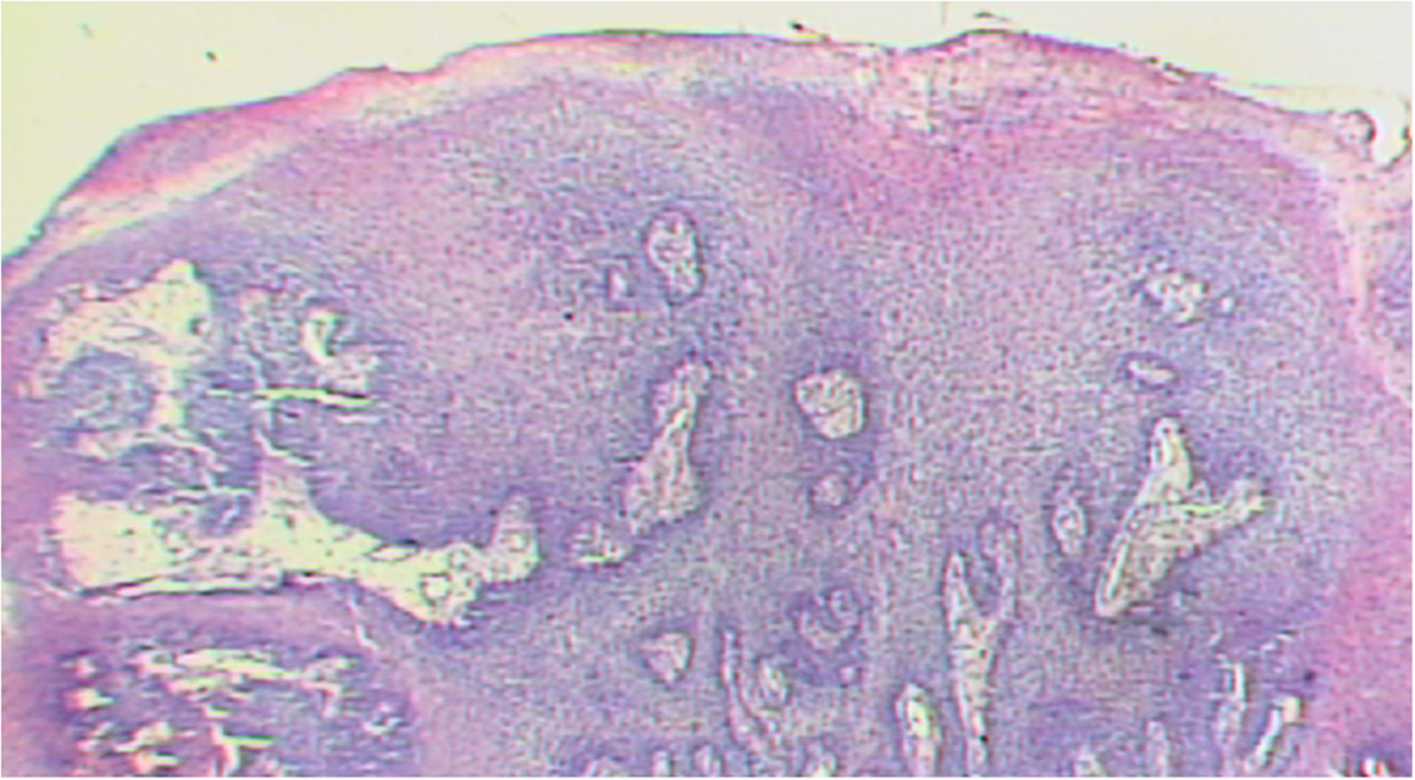
Histopathological analysis, X-40.

**Figure 4:**
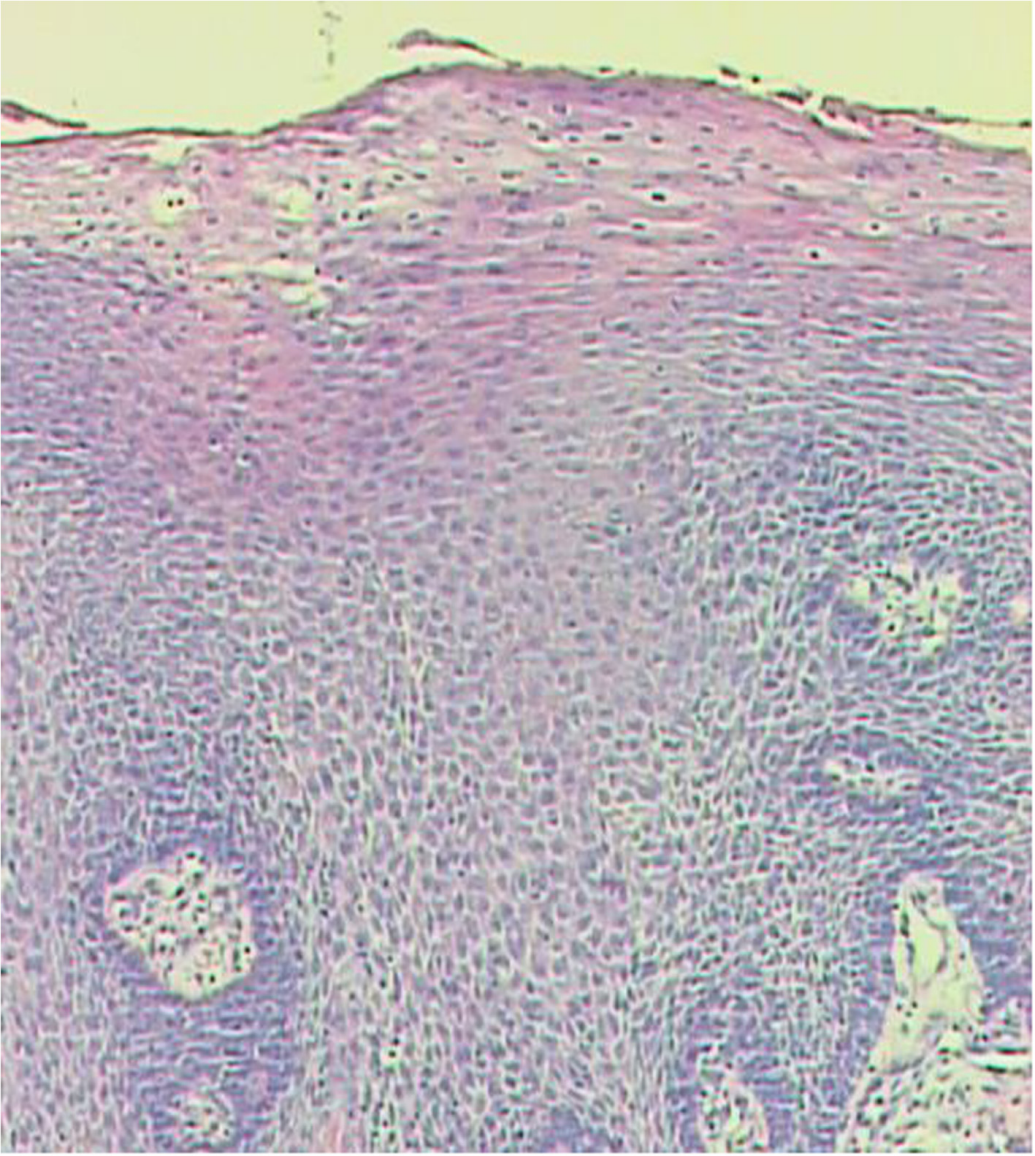
Histopathologically analysis, X-100.

**Figure 5:**
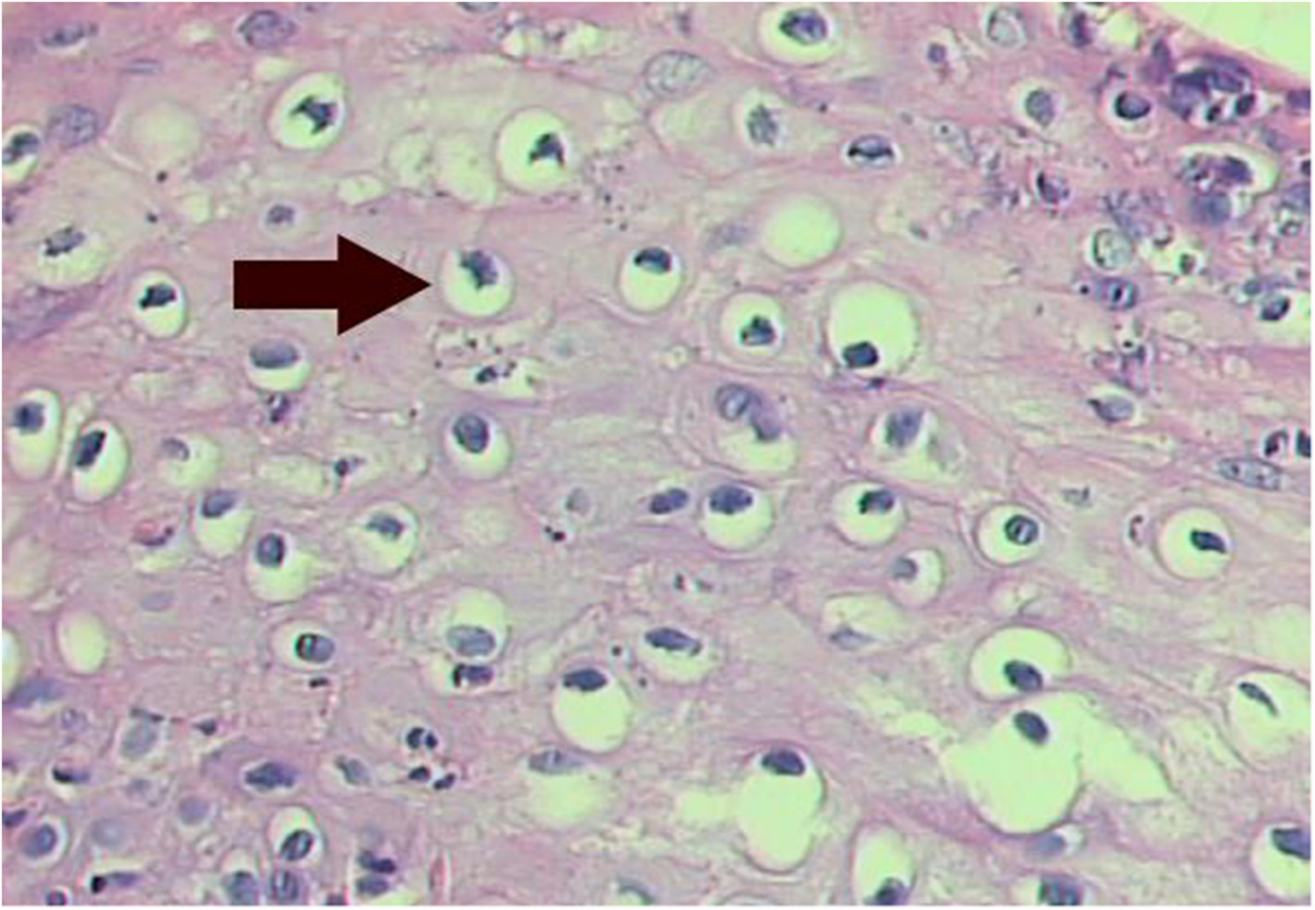
Histopathologically analysis, X-400.koilocytes (arrow).

**Figure 6:**
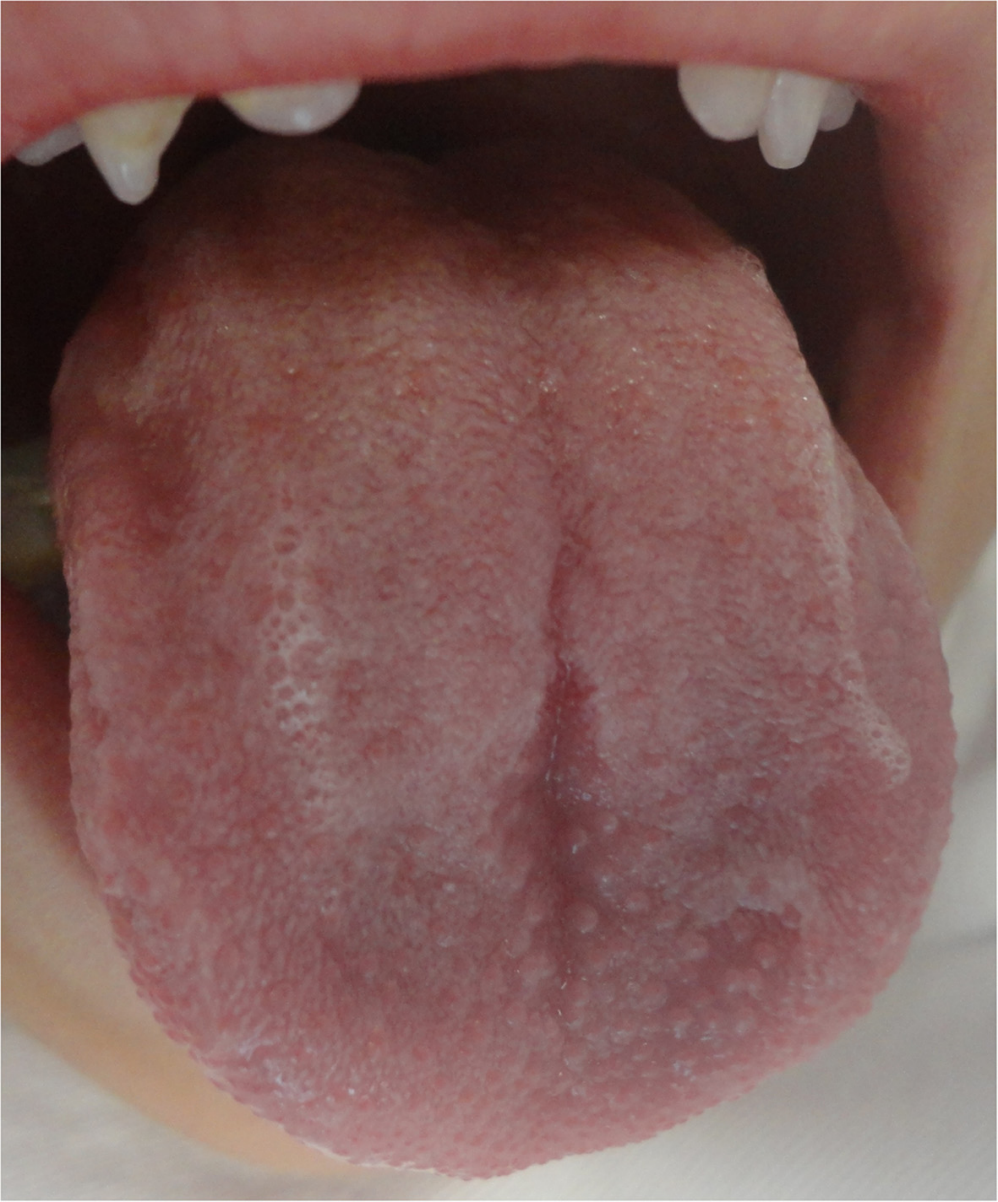
After clinical examination - 30 days (Tongue).

**Figure 7:**
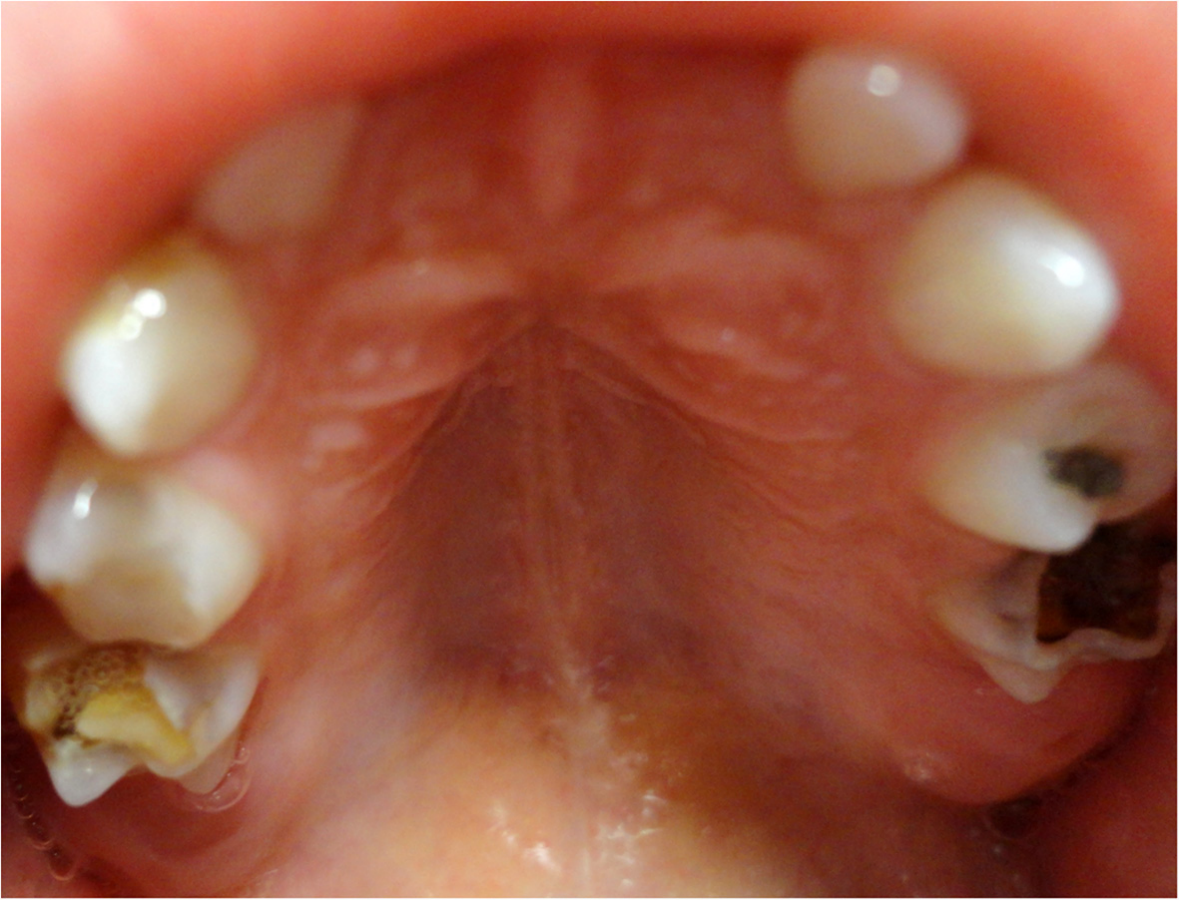
After clinical examination - 30 days (Palate).

The patient, male, five-years-old, Caucasian was referred to the Pediatric Dentistry Clinic, Araçatuba Dental School (Brazil) for an evaluation of a lesion located in the dorsal surface of the posterior tongue. According to the mother’s report, the lesion was observed at the time of oral hygiene and she did not know the precise period of evolution. The patient reported that the lesion was painless, but experienced discomfort during chewing.An intraoral clinical inspection revealed the presence of two lesions. One lesion of approximately a 0.4 mm diameter with a pedicle base, and another one, non-reported by the mother, located in the posterior area of the hard palate with a sessile base with approximately a 0.6 mm diameter (Figures 1 and 2). Both lesions had a firm consistency, reddish appearance and presence of whitish areas and regions of ulceration.During the interview, the mother reported that the boy had been sexually abused by a neighbor, and did not know if these lesions appeared before this act. Excisional biopsies were performed to surgically remove the lesions and they were sent for histopathological analysis. Histopathologically, the biopsy showed acanthotic parakeratinized stratified squamous epithelium containing small parts of conjunctive tissue and koilocytes containing nuclei with a “raisin type” format in the upper spinous layer (Figure 3-40X, Figure 4-X100 and Figure 5-X400). These findings were consistent with condyloma acuminata. After thirty days, the patient returned for a clinical evaluation (Figures 6 and 7).

## Discussion

Condyloma acuminata has been reported to affect the mucosa of the gingiva, cheeks, lips, and hard palate (Figures 1 and 2). A diagnosis of condyloma acuminata is based primarily on the clinical appearance of the lesions and histologic findings in biopsied tissue specimens (Figures 4 and 5 koilocytes (arrow)). Small pink warts may be confused with the lesions. Different types of epithelial hyperplasias, including the condyloma, may look similar within the oral cavity. On the basis of histologic appearance, oral warts could be distinguished from verrucous carcinoma, verruca vulgaris, focal epithelial hyperplasia, and squamous papilloma [12,13]. The microscopic appearance of condyloma acuminata typically includes composed papillary lesions. The lesion has become an increasingly common sexually transmitted infection. The number of reported cases of genital and, in particular, oral warts has recently risen significantly in the United States [12]. The medical evaluation and management of HPV infection in children are complicated by the long latency period of the virus, different modes of transmission and the absence of a unique and effective therapeutic regimen [14].

Transmission of the HPV in children may occur in several ways: during parturition in an infected mother, from close non-sexual contact with infected caregivers, and from one or more sexual encounters. Since the incubation period after exposure to the virus ranges from one to twenty months (average 2 to 3 months), the mode of transmission in individual cases is often not clear [15]. The locations most frequently affected are: the oral mucosa and gums, the floor of the mouth, tonsils and palate. Since these lesions are usually painless, the patient may remain asymptomatic until full spontaneous regression [16] (Figures 1 and 2).

Some authors consider that the presence of condyloma in children may be an indication that there has been sexual abuse [17]. The incidence of sexual abuse in children has been observed in 10-90% of condyloma cases, and this discrepancy is due to the nature of how the sexual abuse investigation was conducted [18]. It is difficult to know the long term effects of sexual abuse for the child. The seriousness of the outcome appears to be linked with other factors, including the age in which the last molestation occurred, the duration and frequency of the molestation, quality of care and experiences with parents in other matters, and assurance from friends and family members that the victim was not to blame. Early effects include anxiety dreams or nightmares, and excessive response to stress with helplessness, fright, psychosomatic symptoms, and the range of behaviors outlined as possible indicators of abuse [19].

Suspicion of abuse can have significant indirect elements: explanations of the injury found are vague or absent, versions of the facts differ from one moment to another and the history is inconsistent with the physical findings [8,9].

Most patients with oral condyloma are treated with surgery [20], with the lesion being excised or electrocauterized. In the present case, however, the treatment chosen was the excision under local oral anesthesia [20,21]. Regardless of the method utilized, condylomas should be treated because they are contagious and can spread out to other oral surfaces, as in this clinical case.

## Conclusions

Clinical manifestations of infection with HPV in the oral cavity have been little investigated by dentists in relation to infection of this virus in the medical area. Therefore, dentists should be aware of the necessity of recognizing wart-like eruptions in and around the oral cavity and identify the connection of these lesions with child sexual abuse. Knowing that condyloma acuminata in children is related to sexual abuse in most cases, dentists need to be more attentive with regard to the early diagnosis, as well as communicating to the parents and legal agencies, thus preventing the child from continuing to suffer from abuse.

## Consent

Written informed consent was obtained from the patient’s parents for publication of this Case Report and any accompanying images. A copy of the written consent is available for review by the Editor-in-Chief of this journal.

## Competing interest

None of the authors had a financial or personal competing interest in relation to this study. All authors approved the publishing of the manuscript.

## Authors’ contributions

ACCP: Contributed during surgical procedure and manuscript writing. MD: Contributed during surgical procedure and manuscript writing. MMC: Contributed histopathological analysis and case description. RFC: Contributed during surgical procedure and manuscript writing. CP: Contributed during surgical procedure and manuscript writing. All authors read and approved the final manuscript.
